# Myocardial scar in diabetics and non-diabetics with ischemic heart disease as assessed by magnetic resonance imaging

**DOI:** 10.1186/1532-429X-11-S1-P194

**Published:** 2009-01-28

**Authors:** Robert Donnino, Andrew H Nguyen, Steven P Sedlis, Monvadi B Srichai

**Affiliations:** 1 NYU School of Medicine, New York, NY USA; 2 Veterans Affairs NYHHCS New York Campus, New York, NY USA

**Keywords:** Coronary Artery Disease, Ejection Fraction, Left Ventricle, Congestive Heart Failure, Ischemic Heart Disease

## Objective

Our objective was to determine the degree of myocardial scarring in diabetic and non-diabetic patients with ischemic heart disease (IHD).

## Background

Diabetic patients with IHD are more likely to develop congestive heart failure (CHF) than non-diabetics, but the mechanism responsible for this is unclear. Recent evidence suggests that this may *not* be related to increased infarct size with accompanying negative remodeling and systolic dysfunction.

## Methods

We retrospectively evaluated 54 consecutive patients (21 diabetics; 33 non-diabetics; mean age 69.7 ± 8.3 years) who underwent both cardiac magnetic resonance (CMR) imaging and coronary angiography for evaluation of IHD between April 2006 and July 2008. Patients were evaluated for presence of CHF symptoms and degree of coronary artery disease (CAD) (measured angiographically using the modified Duke score). Myocardial scar was measured by late gadolinium enhancement on CMR for each patient using a 17-segment model of the left ventricle (LV), and was graded according to transmural extent on a semi-quantitative scale (0 = none, 1 = 1–25%, 2 = 26–50%, 3 = 51–75%, 4 = 76–100%). Total scar burden (mean grade per segment) and spatial extent (number of segments with any scar and number of segments with grades 3–4 scar) were recorded along with LV volume and ejection fraction.

## Results

More diabetics than non-diabetics had CHF symptoms (76.2% versus 45.4%, p < 0.05), and diabetics had a greater burden of CAD (Duke score 4.8 ± 0.7 versus 4.1 ± 1.0, p < 0.05). Diabetics and non-diabetics did not differ, however, in total scar burden (0.92 ± 0.62 versus 1.14 ± 0.73, p = NS), number of segments with any scar (5.6 ± 3.4 versus 6.7 ± 4.6, p = NS), or grade 3–4 scar (3.7 ± 3.0 versus 4.1 ± 3.1, p = NS). LV ejection fraction (38.9 ± 12.7% versus 39.6 ± 16.0%, p = NS) and end diastolic volume (204 ± 86 ml versus 212 ± 75 ml, p = NS) were similar for both diabetics and non-diabetics, respectively.

## Conclusion

Despite a higher incidence of CHF and a greater burden of CAD in diabetics, there was no significant difference in the amount of myocardial scar, LV size or ejection fraction compared to non-diabetics. These findings are consistent with recent literature suggesting that the higher incidence of CHF in diabetics with IHD may not be due to larger infarcts with negative remodeling and systolic dysfunction, but rather to diastolic dysfunction or other factors yet to be accounted for.

